# A simple optimization approach for improving target dose homogeneity in intensity-modulated radiotherapy for sinonasal cancer

**DOI:** 10.1038/srep15361

**Published:** 2015-10-26

**Authors:** Jia-Yang Lu, Ji-Yong Zhang, Mei Li, Michael Lok-Man Cheung, Yang-Kang Li, Jing Zheng, Bao-Tian Huang, Wu-Zhe Zhang

**Affiliations:** 1 Department of Radiation Oncology, Cancer Hospital of Shantou University Medical College, Shantou 515000, Guangdong, China; 2 Department of Clinical Oncology, Prince of Wales Hospital, Shatin, Hong Kong 999077, China; 3 Department of Radiology, Cancer Hospital of Shantou University Medical College, Shantou 515000, Guangdong, China; 4 Department of Laboratory, Shantou Central Hospital, Affiliated Shantou Hospital of Sun Yat-sen University, Shantou 515000, Guangdong, China

## Abstract

Homogeneous target dose distribution in intensity-modulated radiotherapy (IMRT) for sinonasal cancer (SNC) is challenging to achieve. To solve this problem, we established and evaluated a basal-dose-compensation (BDC) optimization approach, in which the treatment plan is further optimized based on the initial plans. Generally acceptable initial IMRT plans for thirteen patients were created and further optimized individually by (1) the BDC approach and (2) a local-dose-control (LDC) approach, in which the initial plan is further optimized by addressing hot and cold spots. We compared the plan qualities, total planning time and monitor units (MUs) among the initial, BDC, LDC IMRT plans and volumetric modulated arc therapy (VMAT) plans. The BDC approach provided significantly superior dose homogeneity/conformity by 23%–48%/6%–9% compared with both the initial and LDC IMRT plans, as well as reduced doses to the organs at risk (OARs) by up to 18%, with acceptable MU numbers. Compared with VMAT, BDC IMRT yielded superior homogeneity, inferior conformity and comparable overall OAR sparing. The planning of BDC, LDC IMRT and VMAT required 30, 59 and 58 minutes on average, respectively. Our results indicated that the BDC optimization approach can achieve significantly better dose distributions with shorter planning time in the IMRT for SNC.

Malignancies of the nasal cavity and paranasal sinuses (sinonasal cancer, SNC) are relatively rare and usually presented with locally advanced disease by the time of diagnosis^1^,^2^,^3^. Surgical resection combined with postoperative radiotherapy can improve the locoregional control and overall survival rates^4^,^5^. Conventional radiotherapy often leads to radiation-induced blindness, retinopathy and optic neuropathy by up to 37%, 40% and 47% of patients, respectively owing to the special anatomy with close proximity of several critical structures such as lenses, eyes, optic nerves, optic chiasm and brainstem^6^. Several reports have demonstrated that intensity-modulated radiotherapy (IMRT) for SNC resulted in significantly reduced ocular toxicity and other side effects while maintaining disease control and survival at least^6^,^7^,^8^.

However, the IMRT planning for SNC is challenging. Besides the close proximity to the nearby critical structures, the presence of the dose inhomogeneity in this particular site also complicates the treatment planning^9^. The target volume typically contains large volumes of air cavities and the buildup region, in which the optimization-convergence error (OCE)^10^,^11^ is extraordinarily significant. The OCE can result in dose discrepancy between the optimizer plans and the finally-calculated plans thus leading to locally high doses (hot spots) or locally low doses (cold spots). The OCE is a systematic error which originates from several major sources including tissue heterogeneity, the buildup region, multi-leaf collimator (MLC) modulation and the optimization algorithm, as described by Dogan *et al.*^11^. The OCE is not able to be overcome by designing optimal arrangement and number of beams, by which IMRT plans are usually effectively improved^12^,^13^.

In this study, we proposed an optimization approach referred to as basal dose compensation (BDC), in which an initial IMRT plan was utilized as a base dose plan for compensating for the OCE. We applied it to SNC cases and then evaluated it by comparing with the initial plan and another commonly-used optimization approach named local dose control (LDC)^14^,^15^. Moreover, volumetric modulated arc therapy (VMAT) technique, an advanced IMRT format with continually rotational gantry, which was previously reported to be dosimetrically superior^16^ or comparable^9^ to conventional IMRT technique in SNC cases, was also adopted for further comparison in this study.

## Methods

### Ethics Statement

The protocol was approved by the Ethical Commission of the Cancer Hospital of Shantou University Medical College. Because this was not a treatment-based study, our institutional review board waived the need for written informed consent from the participants. The patient information was anonymized and de-identified to protect patient confidentiality. The methods were carried out in accordance with the approved guidelines.

### Patient characteristics

We retrospectively identified thirteen patients with malignancies of the nasal cavity, maxillary sinus and ethmoid sinus with stages T2–T4, N0 and M0, according to the American Joint Committee on Cancer (AJCC) 2010 7th edition staging criteria. Of the thirteen patients, seven were males and the remaining six were females, with the median age of 61 years (range, 32–65 years). The pathological types and other detailed information are listed in Table 1.

### CT simulation, target and organ-at-risk (OAR) delineation

The patient immobilization was performed using custom-made thermoplastic masks in the supine position. CT datasets with 3-mm slice thickness were acquired using the 16-slice Big Bore Brilliance CT scanner (Philips Medical System Inc., Cleveland, OH). The CT images were subsequently transferred to the Eclipse version 10.0 (Varian Medical System, Palo Alto, CA) treatment planning system for contouring and treatment planning.

All target volumes were delineated by the attending radiation oncologists. Gross tumor volume (GTV) was defined as the visible extent of tumor identified using contrasted CT, MR and positron emission tomography (PET). Clinical target volume (CTV) comprises the primary tumor bed and the zones at risk of harboring microscopic extension. To account for setup errors, potential intrafractional shifts of patients and mechanical inaccuracies, 0.5 cm margins were added to the CTV to form the planning target volume (PTV). The PTV was prescribed a 60-Gy dose (2 Gy/fraction) administered in 30 fractions. The median volume of the PTV was 183 cubic centimeters (cc) with the range of 102–259 cc.

OARs were contoured including the lenses, optic nerves, optic chiasm, eyes, spinal cord, brainstem, temporal lobes, cochleae, pituitary, oral cavity, parotids. The surrounding normal tissue was defined as the body minus the PTV.

### IMRT and VMAT planning

Seven sliding-window fields of 6-MV photons from a TrueBeam (Varian Medical Systems, Palo Alto, USA) linear accelerator were generated for each IMRT plan in Eclipse. Five coplanar fields were set at 260°, 330°, 0°, 30° and 100° gantry angles and two non-coplanar fields were set at 30° and 330° gantry angles with a couch rotation of 90°. The beam arrangement setting was based on the study by Jeong *et al.*^16^, except that the collimator angles of the coplanar fields with gantry angles of 30° and 330° were optimized for each case aiming at shielding the lenses. For each VMAT plan, two coplanar full arcs were adopted^17^ and the collimator angles were set to 30° and 330°, respectively to minimize the tongue-and-groove effect. The IMRT and VMAT optimizations were performed using the Dose Volume Optimizer (DVO version 10.0.28) and Progressive Resolution Optimizer (PRO version 10.0.28) algorithms, respectively. The Anisotropic Analytical Algorithm (AAA, version 10.0.28) was applied for the final dose calculation. The final plans were normalized to insure that 95% of the PTV received the prescribed dose.

According to the International Commission on Radiation Units and Measurements (ICRU) report 83^18^, the notation D_x_ represents the dose that was reached or exceeded in x of the volume. D_2%_ and D_98%_ indicate the near-maximum and near-minimum doses, respectively. In the inverse optimization objectives, the PTV coverage were assigned the highest priorities, followed by the avoidance of overdosing the lenses, optic chiasm and optic nerves, to preserve mono-lateral vision at least^19^, and the other OAR sparing was given the last priority. The planning goals are shown in Table 2.

The planning objectives from a template were applied and adjusted to generate a generally acceptable initial IMRT plan. In the BDC optimization approach, the number of fractions (NOF) of the initial IMRT plan was modified to x% (0 ≤ x < 100) of the total prescribed NOF to generate a “base dose plan” with x% of the total prescribed dose. Then, the base dose plan was duplicated to create a “top dose plan” with (100–x)% of the total prescribed NOF. Afterwards, the top dose plan was further optimized based on the base dose plan using the “base dose plan” function of Eclipse, while maintaining the optimization objectives unmodified. The “base dose plan” function of Eclipse enabled the system to optimize a plan (as top dose plan) while taking another plan (as base dose plan) into account, aiming to achieve an optimal plan sum by making up for inadequacies (hot/cold spots) in the base dose plan. At this point, the prescribed dose of the plan sum (the base dose plan plus the top dose plan) was equivalent to the original prescription dose. After calculating the final dose of the top dose plan, the NOF of top dose plan (NOF_TDP) was restored from (100–x)% to 100% of the prescribed NOF. The top dose plan with 100% of the prescribed NOF was the final treatment plan for delivery. To establish the BDC approach for SNC cases, we needed to identify the optimal value of the x% of the prescribed NOF of base dose plan (NOF_BDP). Our pilot experiment demonstrated that the dose homogeneity became extremely poor when the NOF_BDP was > 25. Therefore, we tested different NOF_BDP, from 1 to 25 in the present study, to find out which was the best NOF_BDP when applying the BDC approach to SNC cases (the corresponding NOF_TDP was equal to 30 minus NOF_BDP). Using the established BDC approach, the initial IMRT plans were improved by further optimization and the final IMRT plans were named BDC IMRT plans.

In the LDC approach^14^,^15^, the initial optimization objects were kept unchanged, and the cold-spot (≤ 100% of prescribed dose, within PTV) and hot-spot (≥110%, 107% or 105% of the prescribed dose) regions were converted into dose-controlling structures and were then assigned additional lower objectives (101–105% of the prescribed dose) and upper objectives (88%–99% of the prescribed dose), respectively for the further optimization processes. When the final plans achieved the planning goals, the LDC IMRT plans were completed.

The VMAT plans were optimized based on the objectives applied in the initial IMRT plans and were further optimized until the clinically acceptable plans were achieved.

A distributed calculation framework (DCF) was adopted to accelerate the final dose calculation. The total treatment planning time, which accounted for the initial planning time, was recorded for the BDC, LDC IMRT and VMAT plans.

### Plan evaluation

According to the ICRU report 83, the homogeneity index (HI), as a measure of the PTV dose homogeneity, was calculated using the following formula (D_50%_ indicates the median dose):


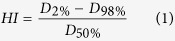


A conformity index (CI)^20^ which takes into consideration the overlap between target volume (TV) and prescription isodose volume (PIV), was used to measure the target dose conformity and was computed using the formula below:


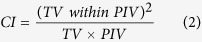


An HI value of 0 stands for the ideal homogeneity, and a CI value of 1 stands for the ideal conformity. D_2%_ was used for evaluating the hot spot, and D_98%_ was used for evaluating the cold spot. The monitor units (MUs) per fraction were also appraised for all plans.

### Statistical analysis

To determine the differences between BDC and initial IMRT, the differences between BDC and LDC IMRT, and the differences between BDC IMRT and VMAT, two-sided paired Wilcoxon signed rank test was used. Data analysis was performed using the SPSS version 19 software (SPSS, Inc., Chicago, IL, USA). Differences were considered to be statistically significant when *P*-value was < 0.05.

## Results

### Establishment of the BDC approach and the HI variation

**Figure 1:**
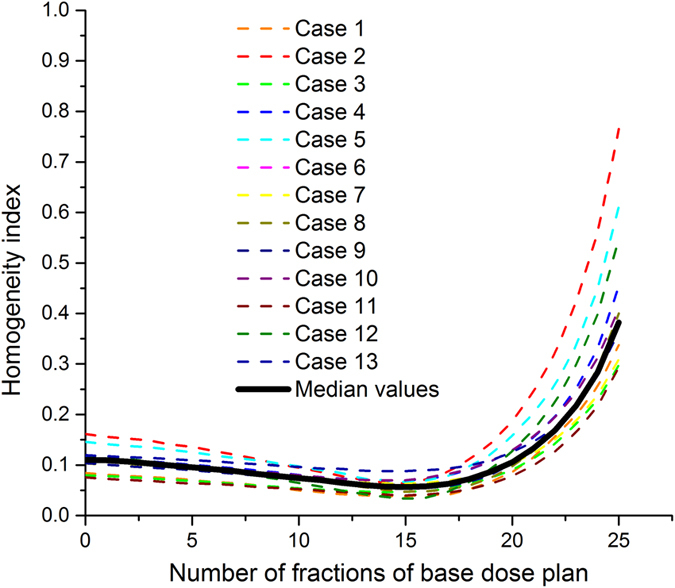
Variation of homogeneity index (HI) under the impact of the number of fractions of base dose plan (NOF_BDP) in the basal-dose-compensation (BDC) optimization approach.

The NOF_BDP indicated the degree of compensation. The HI value of the BDC IMRT plans (optimized top dose plans with 100% of the prescribed NOF) was closely related to the NOF_BDP in the BDC approach. In each case, the NOF_BDP resulting in the lowest HI (indicating the best dose homogeneity) was considered to be the best NOF_BDP. Since the variation tendencies of HIs with the change of NOF_BDP were similar in all cases (see Fig. 1), the frequently-observed best NOF_BDP corresponding to the lowest HI in the greatest number of cases, should be the best one for general use purpose. In our study, the best NOF_BDP values were 15 in 9 cases and 14 in 4 cases, so the 15 (50% of the total prescribed NOF) was determined as the best NOF_BDP for general use in SNC cases.

When the NOF_BDP was 0 (corresponding NOF_TDP was 30), no base dose plan was used for compensation, and the HI value of the BDC IMRT plan was equivalent to that of the initial IMRT plan undoubtedly; the HI decreased slightly (lower HI value indicates better dose homogeneity) approaching the lowest value when the NOF_BDP increased towards approximately 15; when the NOF_BDP was more than approximately 15 and continued to increase towards the prescribed NOF (the NOF_TDP was less than approximately 15 and continued to decrease), the HI increased faster and faster towards the positive infinity, indicating that the dose homogeneity became worse and worse.

In general, the HI was sensitive to the choice of NOF_BDP. When the NOF_BDP value was selected between 14 and 16, low sensitivity with only < 3% difference from the lowest HI was observed; when the NOF_BDP value was between 0 and 13, median sensitivity with 8%–96% difference from the lowest HI was observed; when the NOF_BDP value was between 17 and 25, high sensitivity with 12%–579% difference was observed.

### Target dose homogeneity and conformity

**Figure 2:**
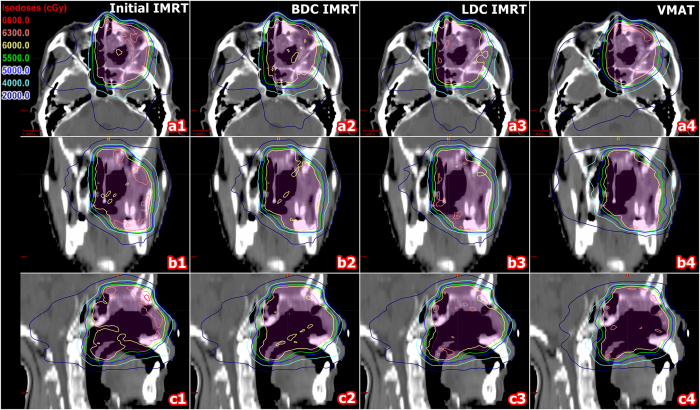
Dose distributions of the initial (a1,b1,c1), basal-dose-compensation (BDC) (a2,b2,c2), local-dose-control (LDC) (a3,b3,c3) intensity-modulated radiotherapy (IMRT) plans and volumetric modulated arc therapy (VMAT) (a4,b4,c4) plans for one representative case.

**Figure 3:**
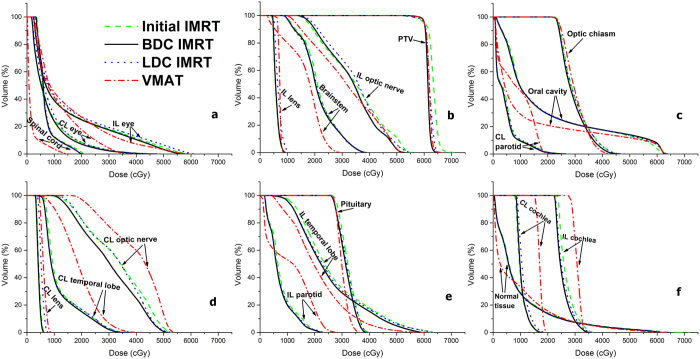
Dose-volume histograms (DVHs) of the initial, basal-dose-compensation (BDC), local-dose-control (LDC) intensity-modulated radiotherapy (IMRT) plans and volumetric modulated arc therapy (VMAT) plans for one representative case. The charts show the DVHs for spinal cord, contralateral (CL) eye, ipsilateral (IL) eye (**a**), IL lens, brainstem, IL optic nerve, planning target volume (PTV) (**b**), CL parotid, oral cavity, optic chiasm (**c**), CL lens, CL temporal lobe, CL optic nerve (**d**), IL parotid, IL temporal lobe, pituitary (**e**), normal tissue, CL cochlea and IL cochlea (**f**).

Table 3 presents the PTV dose-volume parameters for the four plans. The BDC IMRT provided the best homogeneity and satisfactory conformity. With regards to the HI, BDC IMRT was significantly better than initial, LDC IMRT and VMAT by 48.2% ± 11.8%, 23.3% ± 16.1% and 23.0% ± 9.1%, respectively. With regards to the hot and cold spots, BDC IMRT provided lower D_2%_ values by approximately 1.1%–4.1% and higher D_98%_ values by 0.6%–1.3%. Significantly fewer hot spots of ≥ 105% (63 Gy) of the prescribed dose for the PTV were shown in the isodose distributions for BDC IMRT (Fig. 2), which also had a steeper dose-volume histogram (DVH) curve of the PTV (Fig. 3). In terms of the CI, BDC IMRT were superior to initial, LDC IMRT by 9.2% ± 3.9% and 5.5% ± 2.4%, respectively, but inferior to VMAT by 3.2% ± 1.9%.

### OAR sparing

Table 3 summarizes the dose-volume parameters of OARs for the four plans. Compared with the initial and LDC IMRT, the BDC IMRT tended to deposit slightly lower doses to the OARs, and most of the *P* values were < 0.05. BDC IMRT reduced D_2%_ to the bilateral optic nerves and eyes, by approximately 1.7%–3.4% compared with initial IMRT and by 4.4%–7.7% compared with LDC IMRT. Besides, BDC IMRT had similar D_2%_ to the bilateral lenses and optic chiasm compared with initial IMRT, whereas BDC IMRT demonstrated lower D_2%_ to the contralateral lens, ipsilateral lens and optic chiasm by 17.7%, 13.5% and 5.2%, respectively compared with LDC IMRT. Concerning the bilateral temporal lobes, BDC IMRT yielded 1.3%–3.0% lower D_2%_ than initial IMRT, and were comparable to LDC IMRT. In addition, BDC IMRT delivered lower doses to the cochleae by up to 4.6%, and also delivered lower doses to the oral cavity, parotids and normal tissue by 1.7%–6.7%.

When compared with VMAT, BDC IMRT allowed significant reductions of the D_2%_ to the contralateral lens, contralateral- and ipsilateral optic nerve by 14.8%, 11.5% and 2.7%, respectively, and significant reductions of the D_mean_ to the contralateral cochlea, D_50%_ and D_mean_ of the contralateral parotid and D_mean_ to the ipsilateral parotid by 29.4%, 30.2%, 31.5% and 13.1%, respectively. However, BDC IMRT gave higher D_2%_ values to the spinal cord, brainstem and ipsilateral temporal lobe by 100.3%, 34.7% and 10.3%, respectively, and higher D_50%_ and D_mean_ to the oral cavity, higher D_50%_ and D_mean_ of the normal tissue, by 177%, 37.3%, 123.3% and 6.1%, respectively. With regards to the doses to the ipsilateral lens, optic chiasm, bilateral eyes, contralateral temporal lobe, pituitary, ipsilateral cochlea and the D_5%_ to the contralateral cochlea as well as the D_50%_ to the ipsilateral parotid, no statistically significant differences were observed between BDC IMRT and VMAT.

### Planning time and MUs

The average planning time of BDC IMRT was 30.3 minutes, which was less than those of LDC IMRT and VMAT by 41.9% and 46.5%, respectively. The MUs of BDC IMRT were higher than those of initial IMRT and VMAT by 10.7% and 134.6%, respectively, but were lower than those of LDC IMRT by 14.2%.

## Discussion

The achievement of uniform dose distribution of PTV and the sparing of the nearby critical structures, especially the optic structures, were challenging in SNC cases even treated with IMRT. In this study, a BDC optimization approach was well established, and its dosimetric characteristics were evaluated. We found that this approach could substantially improve the dose homogeneity of the target, while improving the target conformity and OAR sparing, thus it may increase the therapeutic ratio for SNC patients. Additionally, this approach could achieve comparable overall results to the VMAT delivery technique.

The improvement of HI by approximately 23%–48% with the established BDC approach may have a potential clinical benefit for SNC patients. Underdosage within the target volume may lead to the likelihood of tumor recurrence. Overdosage within or out of the target volume, may lead to severe acute reactions or late complications, because the target volume of SNC typically contains such tissues as the mucosa, submucosa, nerves and bone^21^. Thus, the improvement of homogeneity may reduce the risk of tumor recurrence and reduce the unnecessary radiation-induced toxicity caused by hot-spot dose.

Moreover, the BDC approach demonstrated improvements with respect to the target conformity and OAR sparing. The BDC approach can reduce the doses delivered to the lenses, optic nerves and chiasm as well as eyes by up to 18%. Although Duprez *et al.*^6^ have reported that the IMRT technique could minimize ocular toxicity, such as blindness and optic neuropathy, compared with conventional radiotherapy technique, there were still 10 cases of late Grade 3 tearing and 1 case of late Grade 3 visual impairment in 86 patients available for late toxicity evaluation. Therefore it is necessary to develop the IMRT technique to further minimize the dose delivered to the optic pathways. Besides, Bhandare *et al.*^22^ observed that the sensorineural hearing loss is significantly correlated with dose to cochlea, and our data showed that the BDC approach can reduce the dose to cochlea by up to 5% and may thus reduce the risk of sensorineural hearing loss. Moreover, the BDC approach can reduce the doses to the temporal lobes, oral cavity and parotids in varying degrees, so it may have the potential to lower the risks of temporal lobe injury^23^, oral mucositis^24^ and xerostomia^25^,^26^.

Another superiority of the BDC optimization approach lies in its shorter planning time. The explanation is that an excellent homogeneous dose distribution can be effortlessly achieved via a single further optimization and the required procedure of modifying one parameter (NOF_BDP) is very simple. Reduction of the planning time is beneficial for reducing the time that patients must wait until the start of treatment and thus for relieving patients’ anxieties. By contrast, the LDC approach is time-consuming because multiple further optimizations are usually required, and additionally, the delineation of the dose-controlling structures and the assignment and adjustment of new dose objectives usually take a certain amount of time. On the other hand, the VMAT planning time is also longer compared to BDC IMRT because the calculating process of AAA and optimizing process of PRO is time-consuming. With the progress of algorithms, VMAT planning time would decrease in the future.

Although the defect of the BDC approach lies in the increasing of the MUs by 11% compared with the initial IMRT, the MUs were still lower than those of LDC IMRT. VMAT had the advantage of significantly lower MUs and normal-tissue dose. Considering the higher doses to normal tissue and the excessive MUs may increase the risk of radiation-induced secondary malignancies^27^ in theory, the VMAT technique is superior in this situation.

Conventionally, planers employ the base dose function to optimize a second-course plan (as top dose plan), e.g., a boost plan, based on the first-course plan (as base dose plan), to achieve an optimal plan sum (top dose plan plus base dose plan) in the optimizer. However, in the BDC approach, the base dose function is used in a new way; here, it is adopted to achieve a homogeneous-dose plan (top dose plan) in the finally-calculated version instead of a plan sum in the optimizer. In principle, the base dose function is utilized to compensate for the OCE. If the OCE introduces a hot spot into the finally-calculated dose of the initial plan (base dose plan), the BDC plan (top dose plan) will generate a cold spot in the same region to obtain a homogeneous summed dose. After final dose calculation of the BDC plan (top dose plan), the OCE introduces a hot spot into the cold-spot region of the BDC plan (top dose plan), and as a result, the final BDC plan will achieve a uniform dose.

Numerous studies have focused on the possible approaches or techniques to solve the OCE. The LDC optimization method described by Süss *et al.*^14^ and applied by Xhaferllari *et al.*^15^ is helpful for overcoming the OCE, but only locally effective in the dose-controlling region. It is a “trial and error” approach because planners need to manually adjust the additional objectives. On the contrary, the BDC method is globally effective and is a systematic approach. According to the review by Broderick *et al.*^28^ and other previous studies^29^,^30^, the Direct Aperture Optimization (DAO) technique incorporates series of deliverable MLC shapes instead of ideal intensity maps in the optimizer and is therefore able to eliminate the error contributed by MLC modulation. Unfortunately, this technique is not available in non-DAO treatment planning systems, e.g., Eclipse version 10.0. Verbakel *et al.*^31^ have reduced the error originating from tissue heterogeneity by separating the PTV into low- and relatively high-density regions and subsequently setting a higher dose goal for the low-density region in the optimizer. This method is effective in the lung cancer cases, but it is not effective enough for SNC cases. As tested in our pilot study, a clinically acceptable plan with D_2%_ of PTV < 110% of prescribed dose could not be achieved with this method. Zacarias and Mill^31^ also utilized the base dose function to solve the OCE, but that method is not the same as ours, because it required a complicated process and software and thus resulted in increased planning steps and time. On the contrary, our approach is much simpler and practical for routine use.

The limitation of this study is that only the dosimetric characteristics of the BDC approach were reported. Whether this proposed approach can bring real benefits to SNC patients remains questionable. The actual clinical benefits should be proved by follow-up in our further studies.

## Conclusion

In this study, we established and evaluated a simple optimization method referred to as basal-dose-compensation approach for SNC. We found that this approach can improve the target dose homogeneity, conformity and OAR sparing, with shorter planning time and with acceptable MU number. In addition, it can achieve comparable overall dosimetric results to VMAT. Consequently, the proposed optimization method is recommended for incorporation into routine clinical practice for the IMRT for SNC.

## Additional Information

**How to cite this article**: Lu, J.-Y. *et al.* A simple optimization approach for improving target dose homogeneity in intensity-modulated radiotherapy for sinonasal cancer. *Sci. Rep.* **5**, 15361; doi: 10.1038/srep15361 (2015).

## Figures and Tables

**Table 1:** Patient characteristics.

Case	Gender	Age (years)	Tumor site	Aim of radiotherapy	Pathological type	Stage
1	Male	65	nasal cavity	Postopereative	Melanoma	T3N0M0
2	Male	63	nasal cavity	Postopereative	Melanoma	T2N0M0
3	Male	32	nasal cavity	Postopereative	Esthesioneuroblastoma	T3N0M0
4	Female	61	nasal cavity	Definitive	NK/T cell lymphoma	T3N0M0
5	Male	59	maxillary sinus	Postopereative	Sarcoma	T2N0M0
6	Female	51	nasal cavity	Definitive	NK/T cell lymphoma	T3N0M0
7	Female	65	nasal cavity	Definitive	NK/T cell lymphoma	T3N0M0
8	Male	62	maxillary sinus	Postopereative	SCC	T4N0M0
9	Female	53	nasal cavity	Postopereative	SCC	T4N0M0
10	Female	64	nasal cavity	Postopereative	Esthesioneuroblastoma	T2N0M0
11	Male	50	ethmoid sinus	Postopereative	SCC	T4N0M0
12	Male	65	maxillary sinus	Postopereative	Adenoid cystic carcinoma	T3N0M0
13	Female	51	nasal cavity	Postopereative	Melanoma	T4N0M0

*Abbreviations:* NK = Natural killer; SCC = Squamous cell carcinoma.

**Table 2:** Planning goals for the treatment plans for sinonasal cancer.

Structure	Planning constraint(s)
PTV	D_95%_ = 60 Gy
	D_2%_ < 66 Gy (110% of the prescription dose)
Lens	D_2%_ < 10 Gy
Optic nerve	D_2%_ < 54 Gy
Optic chiasm	D_2%_ < 54 Gy
Eye	D_2%_ < 50 Gy
Spinal cord	D_2%_ < 40 Gy
Brainstem	D_2%_ < 50 Gy
Temporal lobe	D_2%_ < 60 Gy
Cochlea	D_5%_ < 55 Gy; D_mean_ < 45 Gy
Pituitary	D_2%_ < 64 Gy
Oral cavity	D_mean_ < 30 Gy
Parotid	D_50%_ < 30 Gy; D_mean_ < 26 Gy
Normal tissue	As low as possible

*Abbreviation:* PTV =  planning target volume; D_x%_ = dose that is reached or exceeded in x% of the volume; D_mean _= mean dose.

**Table 3:** Dosimetric parameters for the initial, basal-dose-compensation (BDC), local-dose-control (LDC) intensity-modulated radiotherapy (IMRT) plans and volumetric modulated arc therapy (VMAT) plans.

		Initial IMRT	BDC IMRT	LDC IMRT	VMAT	*P* value (BDC IMRT *vs*)		
						Initial IMRT	LDC IMRT	VMAT
PTV	D_2%_ (Gy)	65.56 ± 1.11	62.84 ± 0.74	63.64 ± 0.73	63.52 ± 0.79	0.001	0.001	0.001
	D_98%_ (Gy)	58.63 ± 0.62	59.36 ± 0.25	59.03 ± 0.48	58.98 ± 0.30	0.002	0.060	0.001
	D_50%_ (Gy)	62.31 ± 0.65	61.23 ± 0.29	61.73 ± 0.37	61.87 ± 0.50	0.001	0.001	0.001
	HI	0.111 ± 0.024	0.057 ± 0.015	0.075 ± 0.015	0.073 ± 0.017	0.001	0.002	0.001
	CI	0.795 ± 0.033	0.866 ± 0.016	0.822 ± 0.023	0.895 ± 0.017	0.001	0.001	0.002
CL lens	D_2%_ (Gy)	6.77 ± 1.22	6.74 ± 1.28	8.28 ± 1.73	7.88 ± 0.83	0.594	0.001	0.004
IL lens	D_2%_ (Gy)	8.95 ± 2.11	8.93 ± 2.19	10.65 ± 3.80	8.79 ± 1.47	0.484	0.001	0.507
CL optic nerve	D_2%_ (Gy)	45.42 ± 9.77	43.97 ± 9.82	47.16 ± 12.63	49.28 ± 5.27	0.003	0.006	0.007
IL optic nerve	D_2%_ (Gy)	53.53 ± 3.49	52.00 ± 3.68	56.05 ± 4.77	53.47 ± 3.66	0.028	0.001	0.011
Optic chiasm	D_2%_ (Gy)	46.15 ± 7.00	45.57 ± 6.80	48.58 ± 9.75	44.40 ± 9.92	0.402	0.002	0.552
CL eye	D_2%_ (Gy)	35.55 ± 14.04	34.95 ± 13.96	36.49 ± 14.37	34.50 ± 9.16	0.030	0.001	0.807
IL eye	D_2%_ (Gy)	47.06 ± 4.92	46.04 ± 4.85	49.95 ± 5.44	44.84 ± 4.99	0.004	0.001	0.084
Spinal cord	D_2%_ (Gy)	15.80 ± 5.99	15.46 ± 5.81	15.71 ± 585	10.46 ± 7.17	0.025	0.249	0.002
Brainstem	D_2%_ (Gy)	38.33 ± 4.77	38.24 ± 4.91	38.58 ± 4.92	29.91 ± 8.45	0.861	0.043	0.002
CL temporal lobe	D_2%_ (Gy)	38.88 ± 10.89	37.50 ± 9.62	37.71 ± 13.32	39.25 ± 8.33	0.019	0.064	0.196
IL temporal lobe	D_2%_ (Gy)	50.67 ± 6.40	49.98 ± 6.13	48.46 ± 13.48	45.50 ± 6.74	0.016	0.116	0.001
CL cochlea	D_5%_ (Gy)	24.61 ± 14.92	24.55 ± 14.90	25.42 ± 15.58	28.76 ± 8.64	0.286	0.002	0.152
	D_mean_ (Gy)	19.06 ± 11.36	18.98 ± 11.25	19.60 ± 11.81	25.74 ± 6.82	0.249	0.002	0.011
IL cochlea	D_5%_ (Gy)	35.10 ± 9.23	34.18 ± 8.80	35.89 ± 9.50	33.43 ± 8.36	0.013	0.003	0.600
	D_mean_ (Gy)	28.31 ± 7.15	27.71 ± 7.29	28.76 ± 7.82	29.35 ± 6.30	0.007	0.004	0.382
Pituitary	D_2%_ (Gy)	44.38 ± 9.33	44.44 ± 9.48	45.34 ± 10.45	41.26 ± 12.19	0.861	0.075	0.087
Oral cavity	D_50%_ (Gy)	16.84 ± 7.07	16.58 ± 6.95	17.25 ± 7.20	8.26 ± 8.70	0.030	0.003	0.004
	D_mean_ (Gy)	22.65 ± 6.24	22.25 ± 6.03	22.84 ± 6.29	16.85 ± 6.45	0.002	0.002	0.001
CL parotid	D_50%_ (Gy)	5.55 ± 6.76	5.33 ± 6.56	5.42 ± 6.51	7.45 ± 7.09	0.002	0.028	0.003
	D_mean_ (Gy)	6.64 ± 5.20	6.39 ± 5.08	6.56 ± 5.10	9.15 ± 5.44	0.001	0.001	0.002
IL parotid	D_50%_ (Gy)	8.48 ± 6.57	8.00 ± 6.36	8.26 ± 6.51	8.98 ± 7.30	0.001	0.003	0.650
	D_mean_ (Gy)	9.55 ± 6.10	9.07 ± 5.91	9.35 ± 6.06	11.00 ± 6.47	0.001	0.001	0.004
Normal tissue	D_50%_ (Gy)	2.82 ± 2.22	2.71 ± 2.14	2.83 ± 2.22	1.37 ± 1.24	0.001	0.001	0.001
	D_mean_ (Gy)	7.49 ± 2.08	7.30 ± 2.02	7.47 ± 2.08	6.90 ± 1.94	0.001	0.001	0.004
Monitor units		862 ± 109	958 ± 152	1157 ± 320	410 ± 19	0.001	0.006	0.001
Planning time	(minute)	NA	30.3 ± 4.3	58.6 ± 23.6	57.6 ± 6.7	NA	0.001	0.001

*Abbreviations:* PTV = planning target volume; CL = contalateral; IL =  ipsilateral; NA =  not applicable; D_x%_ = dose which is reached or exceeded in x% of the volume; HI = homogeneity index; CI =  conformity index; D_mean_ =  mean dose.
